# Supplementary material for: Development of Bisphenol-A-Glycidyl-Methacrylate- and Trimethylolpropane-Triacrylate-Based Stereolithography 3D Printing Materials
Source: Polymers (Basel). 2022 Nov 29;14(23):5198. doi: 10.3390/polym14235198 (PMC9736893; doi:10.3390/polym14235198)
Supplement: Supplementary file 1 [file polymers-14-05198-s001.zip › polymers-2007500-supplementary.pdf]

polymers

Supplementary Materials

Development of Bisphenol-A-Glycidyl-Methacrylate- and Trimethylolpropane-Triacrylate- Based Stereolithography 3D Printing Materials

Yura Choi [1], Jisun Yoon [1], Jinyoung Kim [1], Choongjae Lee [1], Jaesang Oh [2,*] and Namchul Cho [1,*]

1 Department of Energy Systems Engineering, Soonchunhyang University, Asan 31538, Republic of Korea
2 Department of Neurosurgery, College of Medicine, Soonchunhyang University, Asan 31538, Republic of Korea
* Correspondence: metatron1324@hotmail.com (J.O.); chon7@sch.ac.kr (N.C.)

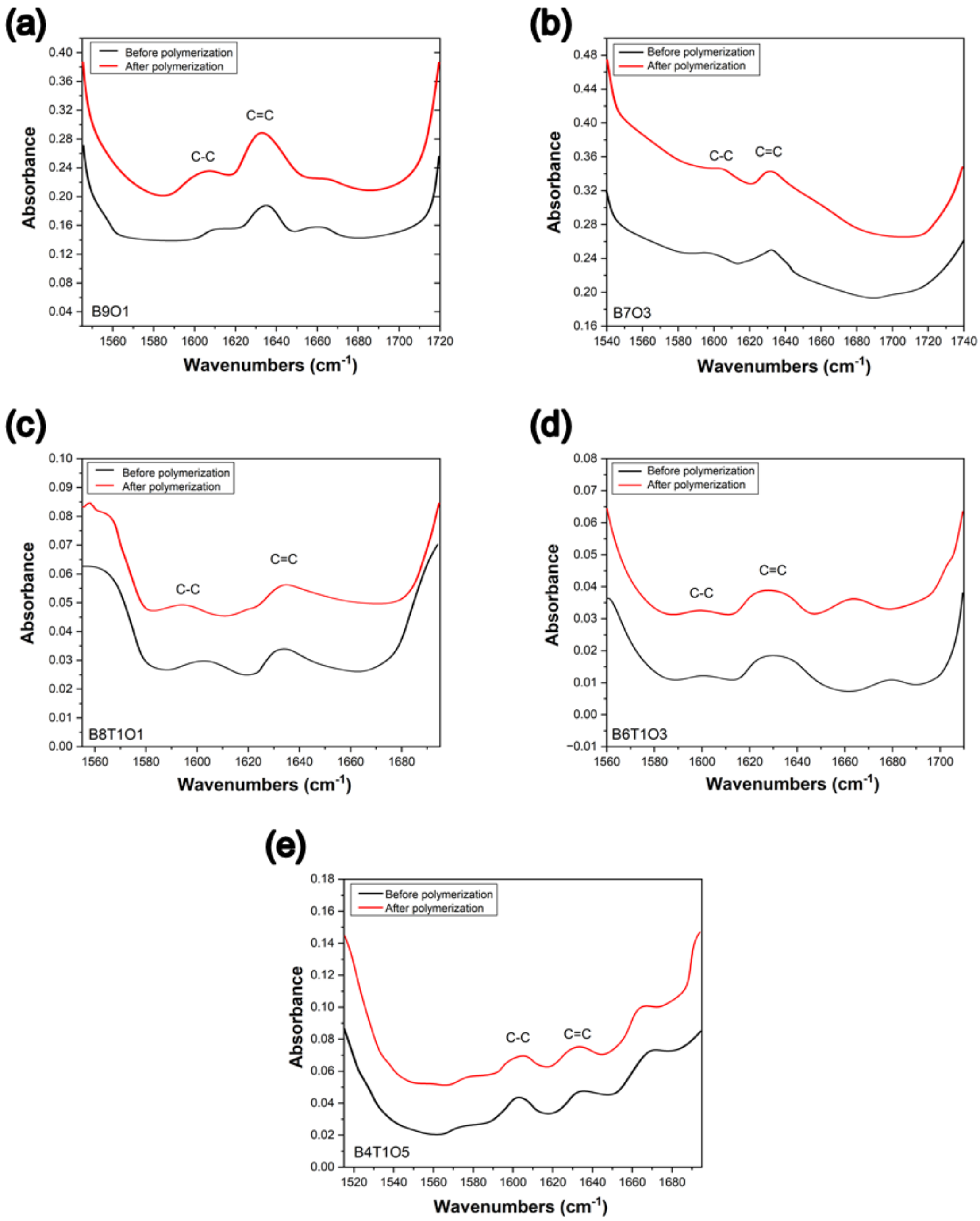

Figure S1. FT-IR spectra before and after polymerization showing typical C–C and C=C peaks for (a) B9O1, (b) B7O3, (c) B8T1O1, (d) B6T1O3, and (e) B4T1O5.
